# Supplementary material for: Ultrafast electron calorimetry uncovers a new long-lived metastable state in 1T-TaSe2 mediated by mode-selective electron-phonon coupling
Source: Sci Adv. 2019 Mar 1;5(3):eaav4449. doi: 10.1126/sciadv.aav4449 (PMC6397029; doi:10.1126/sciadv.aav4449)
Supplement: http://advances.sciencemag.org/cgi/content/full/5/3/eaav4449/DC1 [file supp_5_3_eaav4449__index.html]

Science Advances | Science Advances

## Supplementary Materials

**The PDF file includes:**

- Section S1. Data analysis of trARPES spectra
- Section S2. DFT calculations of electronic structure
- Section S3. Evolution of the electron temperature
- Section S4. The electronic band shift and the new metastable states
- Section S5. Relationship between the band shift and the CDW order
- Section S6. Caption of the supplementary movie
- Fig. S1. Fit of the trARPES spectra.
- Fig. S2. Band structure for 1*T*-TaSe2 in the metallic state (1 × 1) with spin-orbit coupling.
- Fig. S3. Partial density of states projected onto three kinds of Ta atoms in the CDW state ( 13×13) with spin-orbit coupling.
- Fig. S4. Analysis of the electron temperature.
- Fig. S5. Analysis of the band shift.
- Fig. S6. Schematic of the new long-lived metastable state mediated by mode-selective electron-phonon coupling.
- Fig. S7. The long-lasting metastable state.
- Fig. S8. ARPES spectra at selected time delays for the laser fluence of 0.86 mJ/cm2.
- Fig. S9. ARPES spectra at two time delays as a function of laser fluence.
- Legend for movie S1
- References (*46*–*51*)

Download PDF

**Other Supplementary Material for this manuscript includes the following:**

- Movie S1 (.mp4 format). Transforming a material into a new state after heating the electrons with an ultrafast laser.

**Files in this Data Supplement:**

- Adobe PDF - aav4449\_SM.pdf
