# Supplementary material for: Ultrafast electron calorimetry uncovers a new long-lived metastable state in 1T-TaSe2 mediated by mode-selective electron-phonon coupling
Source: Sci Adv. 2019 Mar 1;5(3):eaav4449. doi: 10.1126/sciadv.aav4449 (PMC6397029; doi:10.1126/sciadv.aav4449)
Supplement: Download PDF [file aav4449_SM.pdf]

Science Advances
AAAS

advances.sciencemag.org/cgi/content/full/5/3/eaav4449/DC1

Supplementary Materials for

Ultrafast electron calorimetry uncovers a new long-lived metastable state in 1*T*-$TaSe_2$ mediated by mode-selective electron-phonon coupling

Xun Shi*, Wenjing You, Yingchao Zhang, Zhensheng Tao, Peter M. Oppeneer, Xianxin Wu, Ronny Thomale, Kai Rossnagel, Michael Bauer, Henry Kapteyn, Margaret Murnane

*Corresponding author. Email: xun.shi@colorado.edu

Published 1 March 2019, *Sci. Adv.* **5**, eaav4449 (2019)
DOI: 10.1126/sciadv.aav4449

The PDF file includes:

Section S1. Data analysis of trARPES spectra
Section S2. DFT calculations of electronic structure
Section S3. Evolution of the electron temperature
Section S4. The electronic band shift and the new metastable states
Section S5. Relationship between the band shift and the CDW order
Section S6. Caption of the supplementary movie
Fig. S1. Fit of the trARPES spectra.
Fig. S2. Band structure for 1*T*-$TaSe_2$ in the metallic state (1 × 1) with spin-orbit coupling.
Fig. S3. Partial density of states projected onto three kinds of Ta atoms in the CDW state ($\sqrt{13}\times\sqrt{13}$) with spin-orbit coupling.
Fig. S4. Analysis of the electron temperature.
Fig. S5. Analysis of the band shift.
Fig. S6. Schematic of the new long-lived metastable state mediated by mode-selective electron-phonon coupling.
Fig. S7. The long-lasting metastable state.
Fig. S8. ARPES spectra at selected time delays for the laser fluence of 0.86 mJ/cm^2.
Fig. S9. ARPES spectra at two time delays as a function of laser fluence.
Legend for movie S1
References (*46–51*)

Other Supplementary Material for this manuscript includes the following:

(available at advances.sciencemag.org/cgi/content/full/5/3/eaav4449/DC1)

Movie S1 (.mp4 format). Transforming a material into a new state after heating the electrons with an ultrafast laser.

Section S1. Data analysis of trARPES spectra

We take the data with laser fluence of 0.86 mJ/cm^2 as an example to demonstrate the extraction of the electron temperature and band shift dynamics. Figure S1A displays the temporal evolution of the energy distribution curves (EDC) at the momentum point indicated in Fig. 2A. They are fitted with a Fermi-Dirac function multiplied by a Lorentzian-shape density of states (DOS)

$$I(E) = \left[\frac{1}{e^{(E-E_{\mathrm{F}})/k_{\mathrm{B}}T}+1}\right]\left[\frac{A\Gamma/2}{(E-E_{\mathrm{b}})^2+(\Gamma/2)^2}\right] + I_0 \quad \text{(S1)}$$

where k_{B} is the Boltzmann constant, E_{F} is the Fermi level, T is the temperature, A is an amplitude, E_{b} is the band position, Γ is the width of the Lorentzian function, I_0 is a constant background.

Since we need to fit the EDCs at each delay with the same model and some fitting parameters should share the same value for all data, we implement a global fitting process in the data analysis. Here we discuss three methods. In the first one, we link E_{F}, Γ in each EDC, take T, E_{b}, A, I_0 as independent, and fit all the EDCs simultaneously. From the fitted T and E_{b} at each delay time, we extract the electron temperature and band shift dynamics. Note that since T is influenced by the energy resolution (effectively as T_{res}), we derive electron temperature as $T_e = \sqrt{T^2 - T_{res}^2}$. T_{res} is determined by the fact that $T_e = 300$ K before laser excitation. The results are shown as red circles in fig. S1, B and C. In the second method, we take the width of the Lorentzian Γ as independent for each EDC, considering the possible variation of the imaginary part of the self energy. The results (black circles in fig. S1, B and C) are consistent with that from the first method.

In the third method, we take the DOS as a more generalized form taking the asymmetry of the line shape into account (*46*)

$$I(E) = \left[\frac{1}{e^{(E-E_{\mathrm{F}})/k_{\mathrm{B}}T}+1}\right]\left[\frac{A\cos(\pi\alpha/2+\theta(E-E_{\mathrm{b}}))}{((E-E_{\mathrm{b}})^2+(\Gamma/2)^2)^{(1-\alpha)/2}}\right] + I_0 \qquad \text{(S2)}$$

with

$$\theta(E - E_{\mathrm{b}}) = (1-\alpha)\tan^{-1}\left(\frac{E-E_{\mathrm{b}}}{\Gamma/2}\right) \qquad \text{(S3)}$$

where α is a factor indicating the asymmetry of the DOS. When $\alpha = 0$, there is no asymmetry, Equation (S2) reduces to Equation (S1). The global fitting to the data with this function yields an α value of 0.02, which is very small as seen in fig. S1D. The extracted electron temperature and band shift show good agreements with those from the first two methods.

Based on the above discussions, the first method with a minimal number of fitting parameters provides already good and reliable fits to the EDCs. We apply this method in the data analysis for all laser fluences and the results are discussed in the main text.

We note that the resolution of this calorimetry technique is mainly limited by the energy resolution of the whole instrument, and also depends on the stability of the laser — so that the fluence can be accurately recorded. Based on the data and the statistics presented in Fig. 2, fig. S1 and fig. S4, we estimate the current resolving power of this technique in temperature is about 10, e.g., the resolution is about 100 K when measuring a temperature of 1000 K.

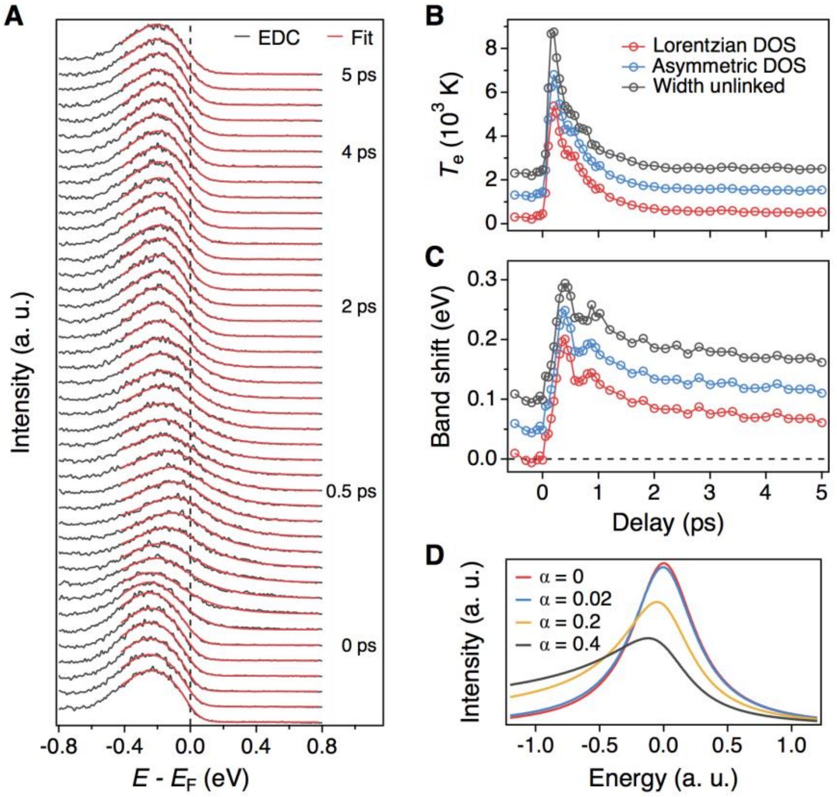

Fig. S1. Fit of the trARPES spectra. (**A**) Time delay evolution of the energy distribution curves. Red curves are fits of the data as described in the supplementary text. (**B** and **C**) Extracted electron temperature and band shift dynamics using different fitting methods. The traces are offset for clarity. (**D**) Comparison of the line shape of DOS with different asymmetries.

Section S2. DFT calculations of electronic structure

Our DFT calculations employ the projector augmented wave (PAW) method encoded in Vienna *ab initio* simulation package (VASP) (*40–42*). The PAW method is used to describe the wavefunctions near the core, and the generalized gradient approximation within the Perdew-Burke-Ernzerhof (PBE) parameterization is employed as the electron exchange-correlation functional (*43*). For band structure calculations, the cutoff energy of 500 eV is taken for expanding the wave functions into plane-wave basis. In the calculation, the Brillouin zone is sampled in the ***k*** space within Monkhorst-Pack scheme (*44*). The spin-orbit coupling is taken into account by the second variation method. In the calculations of 1*T*-$TaSe_2$, the numbers of these ***k*** points are $14 \times 14 \times 8$ and $3 \times 3 \times 5$ for 1×1 unit cell and $\sqrt{13} \times \sqrt{13}$ supercell, respectively. For the CDW phase ($\sqrt{13} \times \sqrt{13}$ cell), we adopt the experimental structural parameters in Ref. *45*.

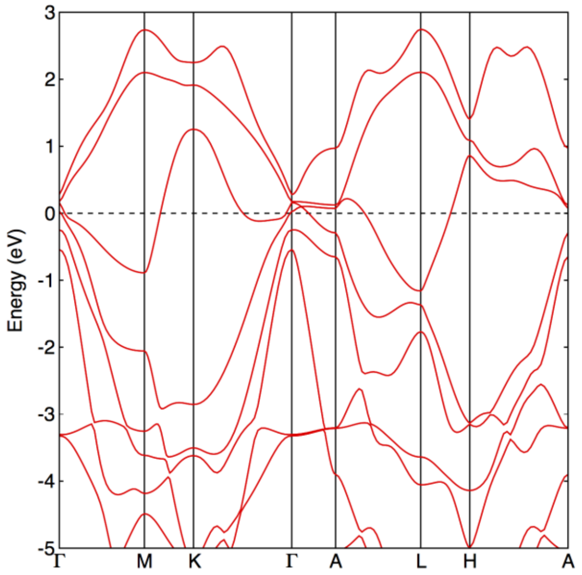

Fig. S2. Band structure for 1*T*-$TaSe_2$ in the metallic state (1 × 1) with spin-orbit coupling.

Figure S2 shows the calculated band structure in the metallic state (1 × 1 cell). In the CDW state, there are three kinds of non-equivalent Ta atoms. We plot the partial density of states projected onto these three Ta atoms in fig. S3. It clearly shows that the electrons tend to "localize" around the center of star-of-David on the occupied side (below E_F), which is consistent with charge density modulation in the CDW state. While on the unoccupied side, there are more states around the outer Ta atoms.

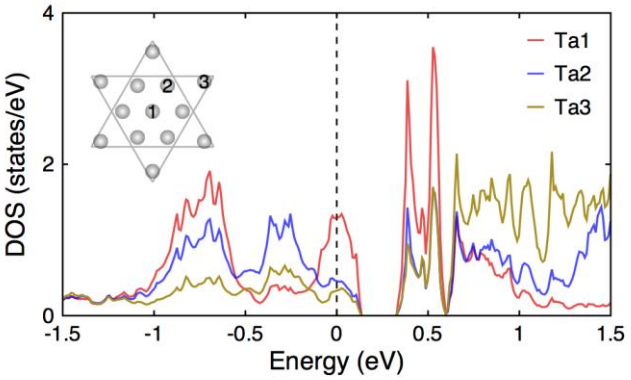

Fig. S3. Partial density of states projected onto three kinds of Ta atoms in the CDW state ($\sqrt{13}\times\sqrt{13}$) with spin-orbit coupling.

Section S3. Evolution of the electron temperature

We fit the electron temperature dynamics by a two-exponential function

$$T_e(\Delta t) = B_1(1 - e^{-g_1\Delta t}) + B_2(e^{-g_2\Delta t} - 1) \quad \text{(S4)}$$

where g_1 and g_2 are the exponential rise and decay constants. The temporal resolution is taken into account by convoluting the fitting function with a Gaussian function (with full width at half maximum of 42 fs). We show the results at two representative laser fluences in fig. S4A. In the laser fluence range shown here, the electron thermalizes on timescales of 20-100 fs (fig. S4B). Interestingly, both of the extracted rise (fig. S4B) and decay constants (fig. S4C) show an anomaly at the critical fluence (F_c, 0.7mJ/cm^2). The change of the rise constant indicates the possible variation of the electron-electron scattering, while the sudden increase of the decay constant suggests the enhancement of the electron-phonon coupling at F_c.

The fluence dependent electron temperature at different time delays are displayed in fig. S4D–F. The maximum of T_e and T_e at 350 fs (the delay around which the band shift is largest) evolve continuously across F_c, while T_e at 4 ps shows a jump of value and slope at F_c, indicating a mode-selective electron-phonon coupling at high fluences as discussed in the main text. Under the condition that the electron system equilibrates with the whole phonon system, the temperature can be approximately estimated from the heat capacity $C(T)$ under thermal equilibrium

$$F(1 - R)/\delta = \int_{T_B}^{T} C(T)\, dT \quad \text{(S5)}$$

with the left term representing the laser energy density at the sample surface (note that ARPES is surface sensitive at this probe photon energy), where R and δ are the reflectance

(~40%) and penetration depth (22 nm), respectively (*6*, *47*). T_B is the sample temperature (300 K) in the experiment. The result is shown as the solid red curve in Fig. 2E and fig. S4F, it captures the fluence dependence of T_e at 4 ps only at $F < F_c$.

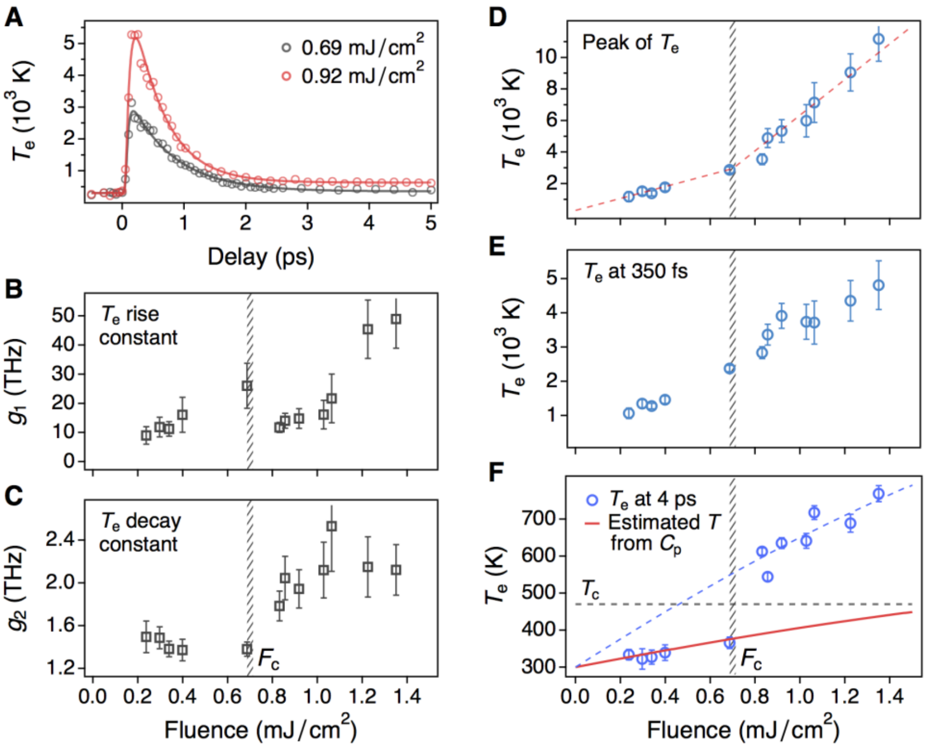

Fig. S4. Analysis of the electron temperature. (**A**) Temporal evolution of T_e at two representative laser fluences. The curves indicate the fits to the data as described in the supplementary text. (**B** and **C**) Extracted rise and decay constant of T_e, respectively. (**D**)-(**F**) The maximum of T_e, T_e at 350 fs and T_e at 4 ps as a function of laser fluence, respectively. The red curve in (F) indicates the temperature estimated from the equilibrium heat capacity.

Note that there is a change of slope (i.e., a kink) in the maximum T_e as a function of fluence (fig. S4D), indicating that the electronic heat capacity at $F < F_c$ is effectively larger than that at $F > F_c$. The enlarged heat capacity at low fluences originates from the energy required to change the state (e.g., the electronic condensation energy or energy for band shift) of 1*T*-$TaSe_2$, which saturates at F_c (orange curve in Fig. 3D). This part of energy corresponds to the latent heat in the first-order transition under thermal equilibrium. Taking this into account, we roughly estimate that the fluence required for the phase transition in an equilibrium way would be ~ 2.2 mJ/cm^2. However, as shown in Fig. 3D and supplementary S5, a significantly

lower fluence of 1.35 mJ/cm^2 is already enough for 1T-$TaSe_2$ to go through and complete the transition in a quasi-equilibrium and metastable way.

Section S4. The electronic band shift and the new metastable states

We fit the EDCs at different momentum cuts around 0.3-0.4 Å^{-1} and extract the band shift, as shown in fig. S5A. The amplitude and dynamics look pretty similar for the results at each $k_{//}$, so it is fair to average them to improve the signal-to-noise ratio. It is worth mentioning that the band shift observed here is clearly different from the chemical potential shift reported in $BaFe_2As_2$ (*48*). Figure S5B shows the temporal evolution of the EDC at $k_{//}$ ~ 0.5 Å^{-1}. We can observe both the dynamics of Ta 5*d* and Se 4*p* bands by optimizing the polarization of the probe beam. The position of Ta 5*d* band (which is the one we discussed in the text unless specified otherwise) exhibits a pronounced shift, while the Se 4*p* band remains nearly unchanged.

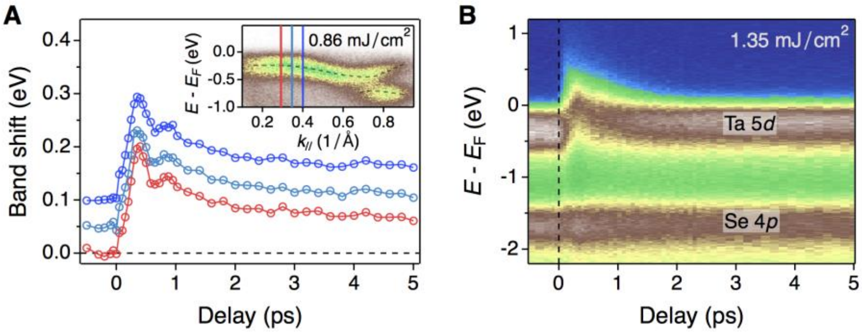

Fig. S5. Analysis of the band shift. (**A**) Extracted band shift dynamics at different momenta as indicated in the inset. The traces are offset for clarity. (**B**) Time-dependent photoemission spectrum at the momentum $k_{//}$ around 0.5 Å^{-1} along the Γ-M direction.

The temporal evolution of the band shift can be modelled by Equations (1) and (2) in the main text, a Gaussian function is convolved during the fitting process to account for the time resolution. Based on the fluence dependence of the fitting parameters, we plot the interpolated two-dimensional band shift dynamics as a function of time delay and laser

fluence in Fig. 4. For $F > F_c$, the material evolves into the new metastable state in several picoseconds, as illustrated in fig. S6.

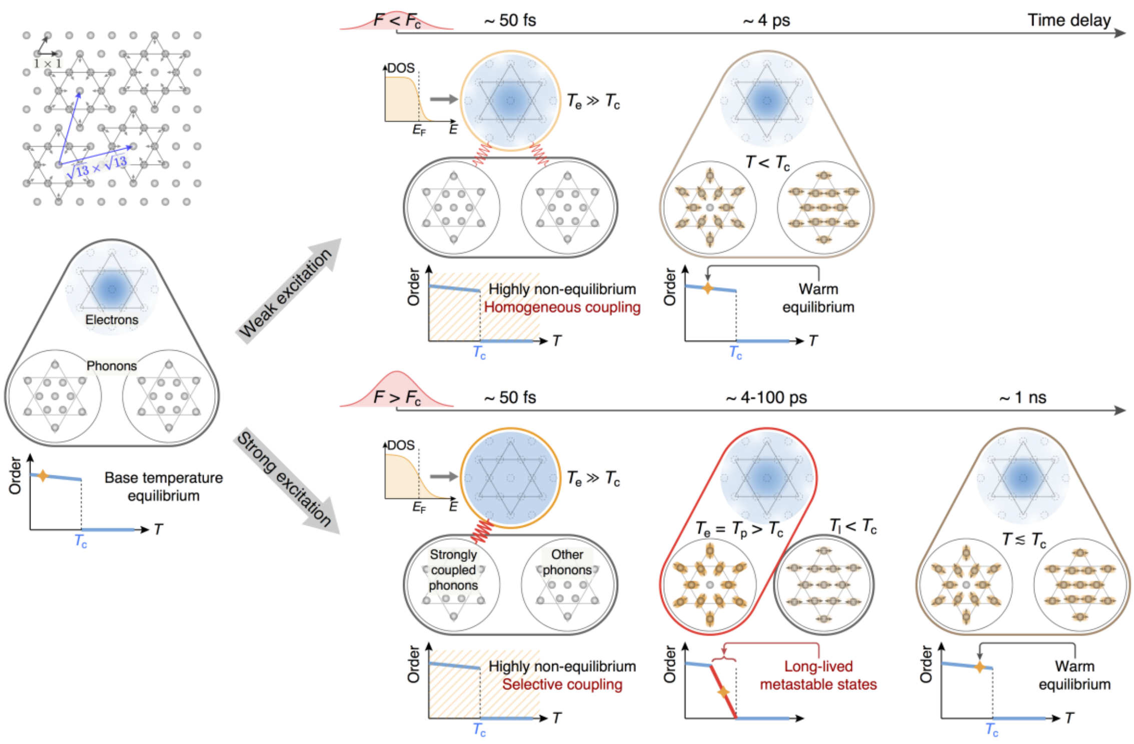

Fig. S6. Schematic of the new long-lived metastable state mediated by mode-selective electron-phonon coupling. Upon laser excitation, the evolution of the sample is determined first by the electron temperature and then by electron-phonon coupling, which are fluence dependent. For strong laser excitation, the electron-phonon coupling switches from nearly homogeneous to mode-selective. The resulting inhomogeneity within the phonon bath drives the material into a new long-lived metastable CDW state. The blue shading represents the electron density in the real-space, the grey circles represent Ta atoms, both amplitudes are exaggerated for better visualization. T_e, T_p and T_l refer to the temperatures of the electron, strongly-coupled phonons, and the rest of the phonon bath, respectively.

This new state lasts for a long time as evidenced by the mild changes of both the band shift and the electron temperature at long time delays. We take the results at the fluence of 0.92 mJ/cm^2 as an example, as shown in fig. S7. For the band shift, the oscillation part is subtracted since it is insignificant in the long-time dynamics. Then we fit the data with a three-exponential function: initial rise, decay to the metastable state (selective electron-phonon coupling) and decay to the quasi-equilibrium state (anharmonic decay of the strongly coupled phonons). The fit yields a timescale of 553 ps for the metastable state to recover. We note that this value is not quantitatively reliable since we only have data before 5 ps, however, it provides the qualitative magnitude of the long lasting time of the new metastable states.

This is further confirmed by the nearly flat part around 4 ps in the electron temperature dynamics (since 1*T*-$TaSe_2$ is a CDW material rather than a simple metal, we avoid using N-temperature model here). It is worth mentioning that this long-lived metastable state and the related timescale are consistent with the results of previous ultrafast electron diffraction experiments (*27*, *49–51*), some of which reported a “second-order character” of the optically induced phase transition but without a clarification of its nature (*27*, *49*).

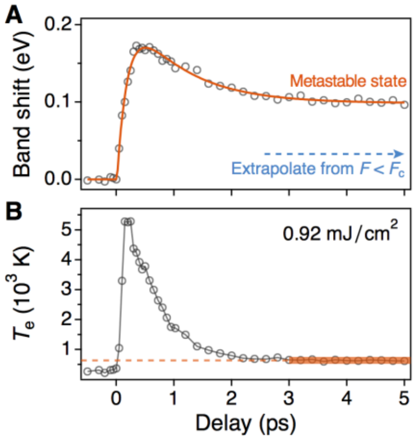

Fig. S7. The long-lasting metastable state. (**A**) The band shift dynamics after subtracting the oscillation part. The metastable state recovers to a quasi-equilibrium state (blue arrow, extrapolate from $F < F_c$) on a timescale of hundreds of picoseconds. (**B**) The electron temperature dynamics that reaches a plateau at several picoseconds.

Section S5. Relationship between the band shift and the CDW order

We observe the coherent oscillation of the electronic band due to the excitation of the CDW amplitude mode. The band shift (Ta 5*d*) is strongly coupled to the atomic displacement in each star-of-David. On the other hand, the characteristic features in the electronic structure of the CDW order, including the band folding and the energy gap, are found to follow the band shift amplitude. This can be clearly seen in the evolution of the ARPES spectrum as a function of both time delay (fig. S8) and laser fluence (fig. S9).

Moreover, we confirm that the observed new metastable state at $F > F_c$ is indeed an CDW ordered state, with the same wave vector as the ground state but with smaller CDW amplitude. As shown in the bottom panels in fig. S9, the spectra in such states show band

folding at the same momentum, but are less pronounced than the normal CDW state. These new states are not reachable under thermal equilibrium conditions, and bridge the first order transition to a continuous one, as illustrated in Figs. 1 and 4.

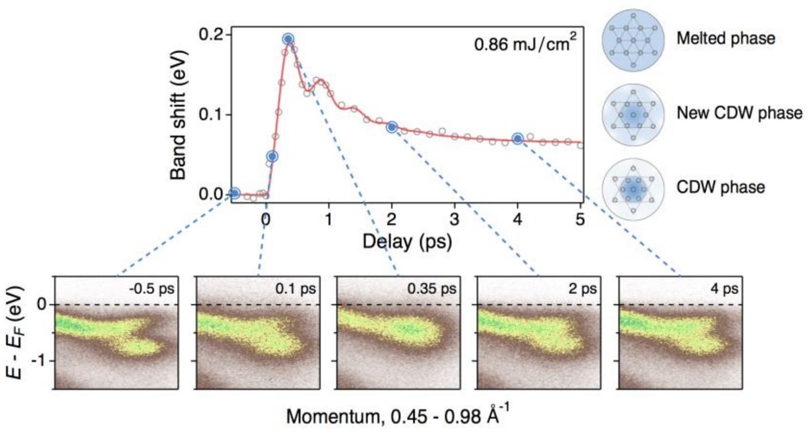

Fig. S8. ARPES spectra at selected time delays for the laser fluence of 0.86 mJ/cm^2.

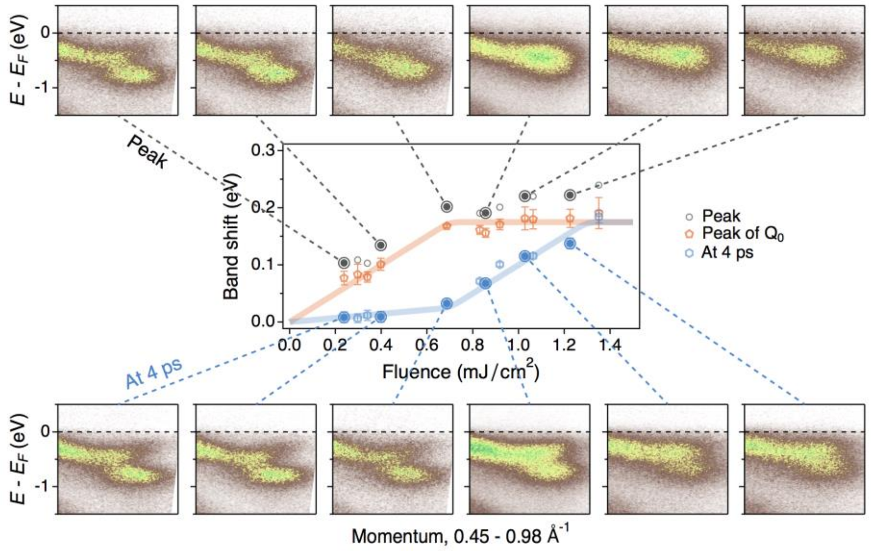

Fig. S9. ARPES spectra at two time delays as a function of laser fluence.

Section S6. Caption of the supplementary movie

Movie S1. Transforming a material into a new state after heating the electrons with an ultrafast laser. Top panel: schematic of the Ta atoms in the 1*T*-$TaSe_2$ crystal lattice (black dots) and electrons (blue shading) in the two phases that can be reached by simply heating the material under thermal equilibrium conditions, e.g., with a hot plate. At low temperatures, the electrons localize (charge order) and the lattice is highly distorted, while at high temperatures, the electrons are smeared and the atomic spacing is uniform. Bottom panel: Movie of the electron energy in the material after rapid heating with a femtosecond laser, i.e., the band structure. The charge order quickly melts, before the material evolves into a new metastable state that lasts for hundreds of picoseconds, characterized by a state with less charge order and lattice distortion. Another remarkable property of this new state is that the electrons are much less coupled to most of the lattice, so that the heat capacity is $\approx$1/3 that of either equilibrium phases. As a result, significantly less energy is required to melt the charge order and transform the state of the material than under thermal equilibrium conditions.
